# Individual Differences in Behavioural Despair Predict Brain GSK-3beta Expression in Mice: The Power of a Modified Swim Test

**DOI:** 10.1155/2016/5098591

**Published:** 2016-07-05

**Authors:** Tatyana Strekalova, Nataliia Markova, Elena Shevtsova, Olga Zubareva, Anastassia Bakhmet, Harry M. Steinbusch, Sergey Bachurin, Klaus-Peter Lesch

**Affiliations:** ^1^Department of Neuroscience, School for Mental Health and Neuroscience, Maastricht University, Universiteitssingel 40, 6229 ER Maastricht, Netherlands; ^2^Laboratory of Biomolecular Screening, Institute of Physiologically Active Compounds, Russian Academy of Sciences, Severnii Proezd 1, Chernogolovka, Moscow 142432, Russia; ^3^Laboratory of Cognitive Dysfunctions, Institute of General Pathology and Pathophysiology, Baltiiskaya Street 8, Moscow 125315, Russia; ^4^Department of Physiology, Federal State Budgetary Scientific Institution “Institute of Experimental Medicine”, Akademika Pavlova Street 12, Saint-Petersburg 197022, Russia; ^5^Department of Human Anatomy, I.M. Sechenov First Moscow State Medical University, Mokhovaya Street 11-10, Moscow 125009, Russia; ^6^Division of Molecular Psychiatry, Laboratory of Translational Neuroscience, Department of Psychiatry, Psychosomatics and Psychotherapy, University of Wuerzburg, Fuechsleinstrasse 15, 97080 Wuerzburg, Germany

## Abstract

While deficient brain plasticity is a well-established pathophysiologic feature of depression, little is known about disorder-associated enhanced cognitive processing. Here, we studied a novel mouse paradigm that potentially models augmented learning of adverse memories during development of a depressive-like state. We used a modification of the classic two-day protocol of a mouse Porsolt test with an additional session occurring on Day 5 following the initial exposure. Unexpectedly, floating behaviour and brain glycogen synthase kinase-3 beta (GSK-3beta) mRNA levels, a factor of synaptic plasticity as well as a marker of distress and depression, were increased during the additional swimming session that was prevented by imipramine. Observed increases of GSK-3beta mRNA in prefrontal cortex during delayed testing session correlated with individual parameters of behavioural despair that was not found in the classic Porsolt test. Repeated swim exposure was accompanied by a lower pGSK-3beta/GSK-3beta ratio. A replacement of the second or the final swim sessions with exposure to the context of testing resulted in increased GSK-3beta mRNA level similar to the effects of swimming, while exclusion of the second testing prevented these changes. Together, our findings implicate the activation of brain GSK-3beta expression in enhanced contextual conditioning of adverse memories, which is associated with an individual susceptibility to a depressive syndrome.

## 1. Introduction 

One of the most critical elements in the pathophysiology of severe clinical depression might entail augmented cognitive processing of adverse events that can aggravate deleterious effects of primarily negative experiences and precipitate the development of stress-related depressive syndromes [1–3]. Currently available data show that enhanced acquisition of fear memories and adverse experiences can be a relevant pathophysiologic mechanism in the development of neuropsychiatric conditions, such as posttraumatic stress disorder, generalized anxiety disorder, phobias, and depression [4, 5]. Nevertheless, the mechanisms underlying reinforced learning of adverse events associated with experience of a specific context during the development of a depressive-like state are poorly understood [6]. The identification of the neurobiology linked to enhanced cognitive processing of aversive experiences would be highly valuable for finding novel therapeutic targets for the treatment of diseases associated with inappropriate retention and processing of memories for environmental adversity.

Animals and humans develop helplessness and fear of environmental contexts in which they have repeatedly experienced aversive events. Most of currently available rodent models of these conditions employ paradigms in which animals learn to associate an aversive unconditioned stimulus, such as a brief footshock, with a neutral conditioned stimulus, typically a context or a tone. In these models, slow extinction learning is usually taken as a measure of inappropriate retention of fear memories and a posttraumatic stress state [7, 8]. While animal models of enhanced learning of adverse challenges including the above-mentioned type of paradigms are well established for phobias, generalized anxiety, and posttraumatic stress disorders, few data are available concerning modeling of cognitive processing of adversities in the development of a depressive syndrome.

Short tests for depressive-like behaviour, such as the Porsolt swim and tail suspension tests, are commonly used in translational research on depression [9–11]. A classical protocol of Porsolt's test is based on the repeated exposure of rodents to an inescapable short swim, an adverse procedure that is typically repeated in the same test condition with a 24 h time interval, when animals display an increase of floating behaviour, commonly referred to as “behavioural despair” [12]. A reexposure of an animal to the originally presented adverse situation is intended to produce a state of (learned) helplessness, one of behavioural hallmarks of depression [12, 13]. Porsolt's test was shown to implicate elements of contextual learning; pharmacologic inhibitors of memory formation were found to attenuate the potentiation of floating during repeated swim sessions [14]. The alleviation of floating in this test by antidepressant drugs was shown to be partly mediated by their diminishing effects on contextual learning [14, 15].

Evidence suggests that with the recollection of stressful and traumatic experiences resulting in the development of depressive-like features, a phase of memory consolidation might be of particular relevance [16, 17]. Based on this, we tested the hypothesis that modification of the classic two-session protocol of Porsolt's test in the mouse, with an additional session occurring on Day 5 following the initial exposure, may potentially model the initiation of consolidation processes that are relevant to the development of a depressive-like state and allow to elucidate their neurobiologic mechanisms experimentally. The timing of the employed protocol of modified swim test was based on available data concerning a time curve of a consolidation of contextual fear memories in respective rodent paradigms, where the primary neuronal processes of a fear consolidation last about 48 h after conditioning and predominantly implicate the hippocampus and prefrontal cortex [18, 19]. We hypothesized that a similar time window might be critical for a formation of a “depressive-like” neurobiological trace related to enhanced cognitive processing of adverse experiences in a modified swim test protocol proposed here. While repeated forced swimming is often applied to induce more pronounced depressive-like phenotypes and helpless behaviour [1, 20, 21], there are no reports available that describe the application of this test for modeling enhanced cognitive processing of adverse experiences.

The aim of the current work was to address the question whether a modified swim test with additional delayed session on Day 5 may model increased contextual consolidation of environmental adversities related to the testing. Therefore, we sought to study whether floating behaviour will be further increased during the delayed swimming on Day 5. In addition, we investigated whether glycogen synthase kinase-3 beta (GSK-3beta) expression is altered in limbic structures during modified swim test protocol. GSK-3beta is a marker of synaptic plasticity in the hippocampus but also of distress and depression. The antibipolar drug lithium and selective GSK-3 inhibitors were reported to increase of GSK-3beta phosphorylation at serine 9 position, thus, decreasing its activity that was associated with beneficial neurobiological and therapeutic effects of these compounds [22–26]. GSK-3beta-dependent functional changes in the hippocampus and prefrontal cortex may influence both anxiety- and depressive-like behaviour, including floating in the forced swim test and cognitive processes [25, 27–33]. Both structures are involved in the consolidation of contextual fear, long-term memories, and enhanced learning during posttraumatic stress disorder [34–37], as well as in the mechanisms of depression [30, 38, 39].

Since we found an increase in both floating behaviour and mRNA GSK-3beta and correlation between them, in the modified swim test, we further investigated the role of exposures of mice to the context of testing versus exposure to the swim test and omission of testing, in the development of above-mentioned molecular and behavioural effects induced in the modified swim paradigm. Also, we addressed the role of timing by carrying out a “premature” testing on Day 3 following the initial swimming instead of a session on Day 5. In addition, we determined the levels of phosphorylated and total GSK-3beta, as well as their ratio, in the hippocampus and prefrontal cortex of mice exposed to the modified swim test. Finally, these behavioural and molecular read-outs were investigated after a fourteen-day treatment with a low dose of the antidepressant imipramine.

Our studies demonstrated the role of contextual elements and timing with adverse experiences of inescapable swimming, in the induction of behavioural and molecular alterations associated with a depressive-like state. They also implicate upregulation of GSK-3beta in the mechanism of enhanced floating response in individual mice that are predisposed to behaviour despair in this modified swim paradigm. This led us to speculate that GSK-3beta, which is both a critical mediator in processes associated with synaptic plasticity and a marker of distress and maladaptation, may play an integrative role in mediating pathologically enhanced memory consolidation for noxious stimulation and a development of associated depressive features.

## 2. Materials and Methods 

### 2.1. Animals

Studies were performed using 3-month-old male C57BL/6J from RAS, Moscow region, a provider licensed by Charles River (http://www.spf-animals.ru/about/providers/animals/). Mice were single-housed under a reversed 12-hour light-dark cycle (lights on: 21:00 h) starting from the day of animals' transportation in the laboratory with food and water provided* ad libitum*, under controllable laboratory conditions (22 ± 1°C, 55% humidity). All experiments were carried out in accordance with the European Communities Council Directive for the care and use of laboratory animals 2010/63/EU and were approved locally (IPAC NM3401165).

### 2.2. Experimental Design

**Figure 1:**
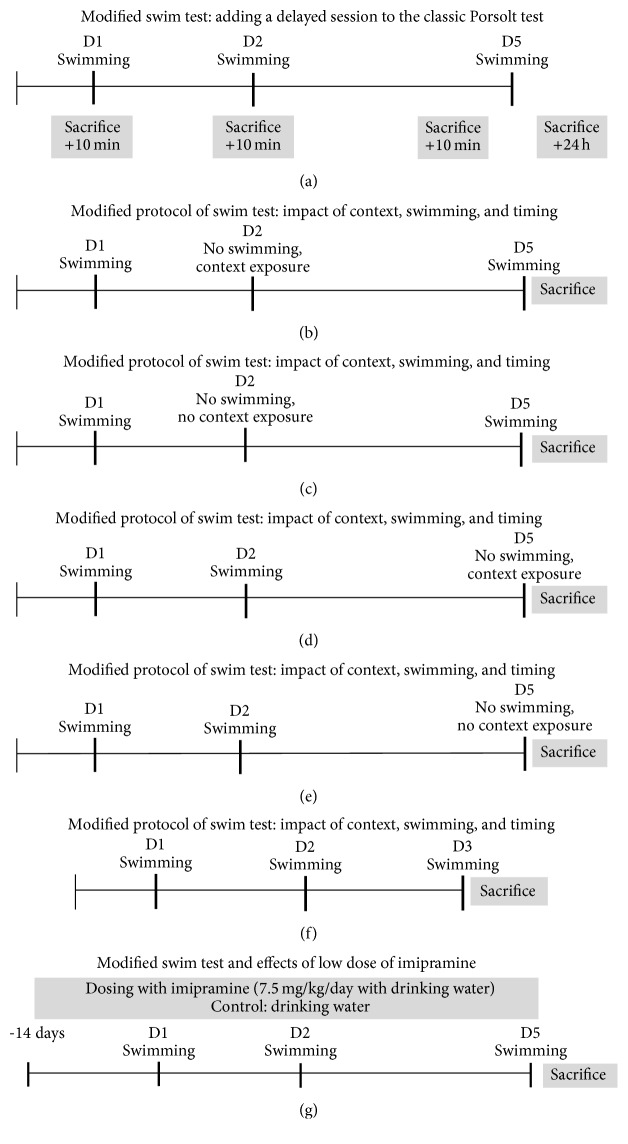
Schematic timeline of experiments. (a) Investigation of behavioural and molecular changes in the modified versus classical swimming paradigms. (b–f) Studies to address the impact of context, swimming, and timing of testing in behavioural and molecular changes induced in the modified swim test. (g) Investigation of behavioural and molecular effects of imipramine in the modified swim test. D1: Day 1; D2: Day 2; D3: Day 3; D5: Day 5.

In the first study we investigated whether in the additional delayed testing in the Porsolt swim test mice display an increase or a decrease of behavioural despair as compared to the classic swim test. Therefore, mice were subjected to the one- and two-session protocols of Porsolt's test, as well as to the modified protocol during which, on Day 5 after initial testing on Day 1, a delayed session was added to a two-day swim test (Figure 1(a)). Their floating behaviour was evaluated in 6-min sessions in 2-min time intervals (see below). The testing was carried out in morning hours, during active period of animal's cycle, in dark laboratory rooms where the apparatuses were lit with subtle lighting (see below). For acclimatization, mice were transported to the experimental room from the lab where they were housed, at least 1 h prior the start of testing. Behaviour was video-recorded and scored offline.

As an increase of floating behaviour on Day 5 was found in above-described experiment, we predicted that this augmentation in behavioural despair is due to cognitive processes taking place during the delay between swim sessions. In the next series of experiments (Figures 1(b)–1(f)) we therefore addressed the individual role of context, swimming, and timing of testing in this phenomenon. Therefore, a replacement of a swim session with an exposure to the context of testing or complete omission of any behavioural manipulation was performed on Day 2 (Figures 1(b) and 1(c)) and on Day 5 (Figures 1(d) and 1(e)). Data were calculated in percent from mean values of floating on Day 1 for each group. Additionally, a group of mice was tested “prematurely” in a three-session swim test where the last test was carried out on Day 3 instead of Day 5 (Figure 1(f)) and compared with a group subjected to the regular protocol of the modified swim test (Figure 1(a)). A study design for this and described below series of experiments is additionally summarized in a Supplementary Tables in Supplementary Material available online at http://dx.doi.org/10.1155/2016/5098591.

In all above-described schemes of testing, mice were sacrificed 10 min after the last behavioural procedure and two brain regions, hippocampus and prefrontal cortex, were dissected for subsequent analyses of GSK-3beta activities using qRT-PCR and ELISA of total and phosphorylated forms of GSK-3beta (see below). While defining a time frame of analysis of GSK-3beta activities, the changes in brain GSK-3beta mRNA at Day 5, the primary experimental setting of interest, were found at the time point 10 min, but not 24 h after testing. Based on these data, and in sake of conformity in the assessments of gene expression and protein changes of GSK-3beta, we compared their measures between all applied experimental schemes of swim test at time point 10 min after the last swim session, unless specified. A group of intact control mice that were naïve for swim experience was used in these assays; each study employed a separate control group. Spearman correlation was performed to link changes in GSK-3beta mRNA concentration to the duration of floating. Additionally, mice were assigned to subgroups designated “high-” and “low-floaters” according to a criterion of “mean of total duration of floating of the group” as that was measured in the swim session preceding a sacrifice of the animal, and the expression of GSK-3beta was compared. Mice which displayed a total duration of floating exceeding the mean of the group at the last testing session were assigned to a subgroup of “high-floaters.” Remaining animals were considered as “low-floaters”; their values of total floating duration measured at the last swim session were below the mean of the group.

Also, in the regular protocol of modified swim test (Figure 1(a)), a subgroup of mice was sacrificed 24 h after the last swimming and compared for GSK-3beta expression with a group sacrificed 10 min thereafter. In addition, in the regular protocol of modified swim test, qRT-PCR of GSK-3alpha was carried out to control for the specificity of changes in GSK-3beta expression.

In the final study, we compared floating behaviour and GSK-3beta mRNA/protein levels in naïve mice and mice pretreated for fourteen days with a low dose of 7.5 mg/kg/day of the antidepressant imipramine (Figure 1(g); see below). The selection of the imipramine dosage was based on previous findings showing absence of confounding effects of this dose on locomotion and weak antidepressant effects in the classic Porsolt swim test in C57BL6 mice [40].

### 2.3. Classic and Modified Swim Tests with Additional Delayed Testing

Mice were subjected to one or two swimming sessions with an interval of 24 h (classic protocols of swim test) or three swim sessions, where the third test was added to a two-day “classic protocol” and carried out on Day 5 following the initial test on Day 1 (modified swim test; Figure 1(a)). All sessions were 6-min long and were performed by placing a mouse in a transparent cylinder (Ø 17 cm) filled with water (+23°C, water height 13 cm, and height of cylinder 20 cm). The duration of floating behaviour that was defined by the absence of any directed movements of the animals' head and body was scored manually using criteria, which were previously validated by automated scoring with Noldus EthoVision XT 8.5 (Noldus Information Technology, Wageningen, Netherlands) and CleverSys (CleverSys, Reston, VA, USA) as described elsewhere [41, 42]. Time spent with floating was evaluated at 2-min intervals as well as summarized for the entire duration of the test. In the experiments with various combinations of swim test protocols, exposures to a context of testing and timing of sacrifice, and imipramine dosing, the total duration of floating was evaluated for the entire 6-min periods. In all studies, except one, which addressed the GSK-3beta expression 24 h after swimming in the modified swim test, mice were sacrificed ten minutes after swimming (see the following).

### 2.4. Exposure to Context of Swimming

To address the role of context in behavioural and molecular changes found in the modified swim test, a series of experiments in which mice were subjected to the context of a swimming test at various time points of testing. Therefore, in the same environment where they were previously subjected to swimming sessions, mice were taken out from the cage by an experimenter and exposed to the swimming pool being hung by their tails and kept above a water surface for 1 sec. Thereafter, they were placed back to their home cages. We chose to apply short-lasting reminding procedure of above-described protocol in order to prevent potential behavioural and molecular aberrations associated with a prolonged suspension of mice by their tails. Based on our previous findings that handling, as an element of this procedure, might be a powerful contextual cue for mice, a sufficient efficacy of selected protocol of reminding procedure was anticipated [43].

### 2.5. Brain Dissection, RNA Extraction, and qRT-PCR

Mice were sacrificed by cervical dislocation as described elsewhere [42] in the experimental area where swim test was performed, except that specific studies were mice supposed to be not exposed to the context of testing prior to sacrifice. The brain of each mouse was dissected and the prefrontal cortex and hippocampi were isolated as previously reported [44–46] and stored at −80°C until use. mRNA was extracted by using TRI Reagent (MRC, Cincinnati, OH, USA). First-strand cDNA synthesis was performed using random primers and Superscript III transcriptase (Invitrogen, Darmstadt, Germany); 1 *μ*g total RNA was converted into cDNA. Quantitative RT-PCR (qRT-PCR) of the GSK-3beta gene and the housekeeping gene glyceraldehyde-3-phosphate dehydrogenase (GAPDH) was performed using the SYBR Green master mix (Bio-Rad Laboratories, Philadelphia, PA, USA) and the CFX96 Real-time System (Bio-Rad Laboratories, Philadelphia, PA, USA). The reference gene was selected in preliminary experiments that demonstrated relatively low variability in GAPDH expression in the limbic structures of the rodents exposed to various stressors, including forced swimming, as compared to the expression of several other housekeeping genes that is in line with other reports [47–49]. Sequences of primers used are listed in Supplementary Material (Supplementary Table 1). Data were normalized to GAPDH mRNA expression and calculated as relative-fold changes compared to control mice as described elsewhere [44].

Results of qRT-PCR measurement were expressed as Ct values, where Ct is defined as the threshold cycle of PCR at which amplified product was 0.05% of normalized maximal signal. We used the comparative Ct method and computed the difference between the expression of the gene of interest and GAPDH expression in each cDNA sample (2^−ΔΔCt^ method). Data are given as expression-fold compared to the mean expression values in nonstressed control mice [44].

### 2.6. ELISA Assays of Total and Phosphorylated Forms of GSK-3beta

The hippocampi and prefrontal cortex were homogenized in buffer containing 10 mM Tris (pH 7.4), 100 mM NaCl, 1 mM EDTA, 1 mM EGTA, 1 mM NaF, 20 mM Na_4_P_2_O_7_, 10% glycerol, and 2 mM Na_3_VO_4_. Protease inhibitor cocktail (Sigma-Aldrich, St. Louis, MO, USA) was added immediately prior to homogenization. The [pS9]-GSK-3beta ELISA kit and [total]-GSK-3beta ELISA kit (Invitrogen Corporation, Carlsbad, CA, USA) were used for detection and quantify the level of GSK-3beta protein phosphorylated at serine residue 9 and total level of GSK-3beta, respectively. All procedures were done according the instruction manual and the optical densities of experimental plates were measured at 450 nm using a plate reader (Wallac 1420 VICTOR, Waltham, MA, USA). The results were normalized to total protein level in tissues homogenates. Protein concentrations were determined by the biuret assay using bovine serum albumin as a standard [50].

### 2.7. Drug Administration

Imipramine (Sigma-Aldrich, St. Louis, MO, USA) was dissolved in tap water; the dose of drug was 7.5 mg/kg and provided for mice to drink* ad libitum* in water bottles for fourteen days prior the beginning of behavioural testing and throughout the entire experiment as described elsewhere [45, 51]. The method of dosing was selected in order to exclude a factor of handling that could be a significant confound of daily intraperitoneal injections or administration of a drug via a gavage with the current model.

### 2.8. Statistical Analysis

Data were analyzed with GraphPad Prism version 5.0 for Windows (San Diego, CA, USA). Paired two-tailed test was used to compare repeated measurements. One-way ANOVA was used to compare three or more groups and two-way ANOVA was applied to analyze “high-” and “low-floaters”; Tukey's* post hoc* multiple comparison test was employed as a* post hoc* test. The trend of the effects of a number of swim sessions on ELISA parameters was analyzed using univariate ANOVA for three groups and* post hoc* test for linear trend. Spearman correlation was used to perform correlational analysis. The level of confidence was set at 95% (*p* < 0.05) and data are shown as mean ± SEM.

## 3. Results

### 3.1. Potentiated Behavioural Despair in the Modified Swim Paradigm

**Figure 2:**
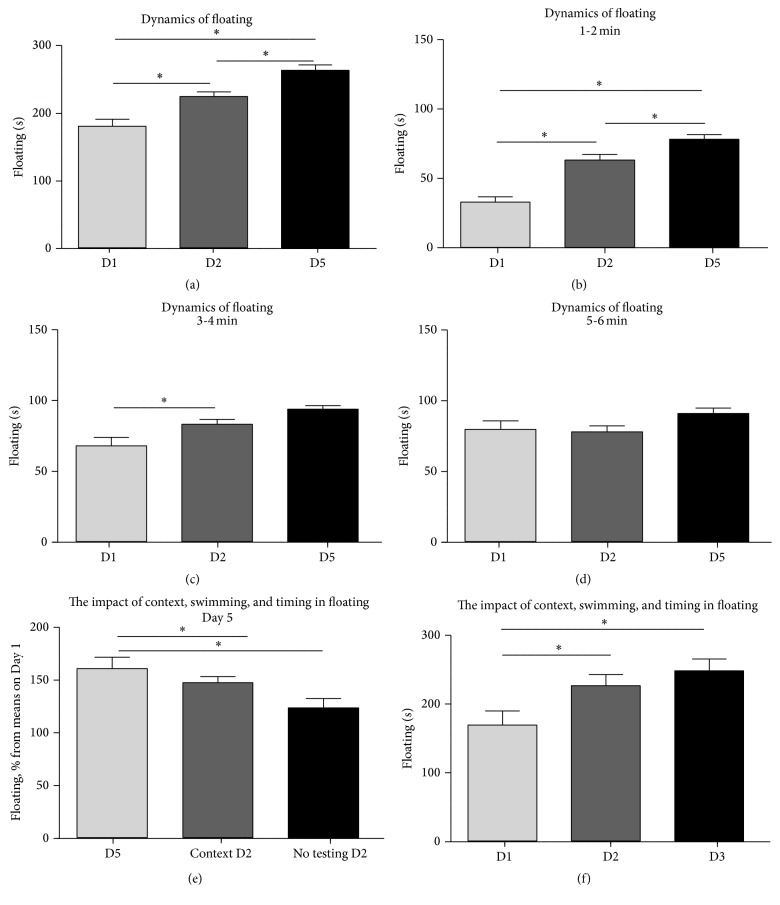
Behavioural changes induced in the modified swim test and the role of context, swimming, and timing of testing. (a) Mice exposed to the modified swim test (*n* = 14) showed an increase of total duration of floating. (b–d) An increase of duration of floating at the delayed testing session on Day 5 in comparison to the session on Day 2 was the pronounced during the first two-minute periods. (e) In comparison to the session on Day 2, mice subjected to the modified swim test have displayed significantly increased total duration of floating when the testing on Day 2 was replaced with exposure to a context of testing (*n* = 10) that was similar to the changes in regular modified swim test, but not when the testing on Day 2 was omitted (*n* = 9). Data are expressed in percent from mean values of floating on Day 1 for each group. (f) Mice displayed a significant increase in the total duration of floating on Day 3 as compared to Day 1, but not Day 2 (*n* = 10). ^*∗*^ *p* < 0.05, ANOVA and Tukey's multiple comparisons test; see the text; Context D2: mice were exposed to a context of testing instead of swim session on Day 2; no testing D2: testing on Day 2 was omitted. D1: Day 1; D2: Day 2; D3: Day 3; D5: Day 5. Data are mean ± SEM.

Repeated measures ANOVA showed significant differences between sessions on Day 1 and Day 2 and Day 2 and Day 5 (*F* = 25.16; *p* < 0.0001, Repeated measures ANOVA summary; Figure 2(a)). While during delayed testing in the modified swim protocol mice could potentially display decreased duration of floating due, for instance, fading away of adverse effect of previous inescapable swimming, repeated pairwise comparison revealed a significant increase of the total duration of floating from Day 2 to Day 3 (*q* = 4.85; *p* = 0.012, Tukey's test; Figure 2(a)). There was a significant increase of this parameter from Day 1 to Day 2 as well (*q* = 5.01; *p* = 0.009, Tukey's test), confirming generally reported data [31]. The increase of despair behaviour at the delayed testing session on Day 5 occurred at the expense of the first two minutes of the test where the duration of floating was sharply elevated as compared to this observation period of the session on Day 2 (*q* = 5.01; *p* = 0.009, Tukey's test; Figures 2(b) and 2(c)). No further enhancement of floating behaviour was found during the second two-minute testing interval and the last two minutes of the swim session as compared to these observation periods on Day 2 (*q* = 3.26; *p* = 0.091 and *q* = 3.32; *p* = 0.085, resp., Tukey's test; Figure 2(d)). An instantaneous increase of floating during testing at the postponed session instead of a gradual increase throughout the testing procedure suggests that the animals were likely to be in a state predisposing to potentiated floating behaviour and displayed this immediately at placement in a pool.

### 3.2. The Role of Context, Swimming, and Timing of Testing in the Potentiation of Floating Behaviour during the Modified Swim Test

Next, in a series of experiments, we addressed the impact of context, swimming, and timing of testing, in the potentiation of floating behaviour during the modified swim test (see Supplementary Table 2). A comparison of groups that were subjected to modified swim test (Figure 1(a)) or similar protocols where the swimming session on Day 2 was replaced with exposure to a context of testing (Figure 1(d)) or omitted (Figure 1(c)) revealed significant differences in the floating duration on Day 5 (*F* = 4.79; *p* = 0.017, ANOVA; Figure 2(e)). Mice from the latter group, but not mice exposed to the context of testing on Day 2, showed a significant decrease in this parameter, as compared with mice exposed to the modified swim test (*q* = 4.31; *p* = 0.014 and *q* = 1.56; *p* = 0.521, resp., Tukey's test; Figure 2(e)). This suggests that exposure to a swim at on Day 2 or a context of testing are crucial for the potentiation of floating on Day 5 and that both have similar impact in this potentiation.

In the next experiment that studied whether enhancement of floating duration during additional session may occur already on Day 3 (Figure 1(f)), repeated measures ANOVA showed significant differences between the days of testing (*F* = 15.52; *p* = 0.0005, repeated measures ANOVA; Figure 2(f)). Pairwise comparison showed that, in comparison to the session on Day 1, the duration of floating in these mice was significantly higher on Day 2 and Day 3, but no difference was found between two latter sessions (*q* = 7.06; *p* = 0.002, *q* = 6.23; *p* = 0.004, and *q* = 2.20; *p* = 0.312, resp., Tukey's test; Figure 2(f)). This suggests that time interval between repeated sessions is a factor of potentiation of floating behaviour in the modified swim test.

### 3.3. The Role of Context, Swimming, and Timing of Testing in the Altered GSK-3beta Brain Expression during the Modified Swim Test

**Figure 3:**
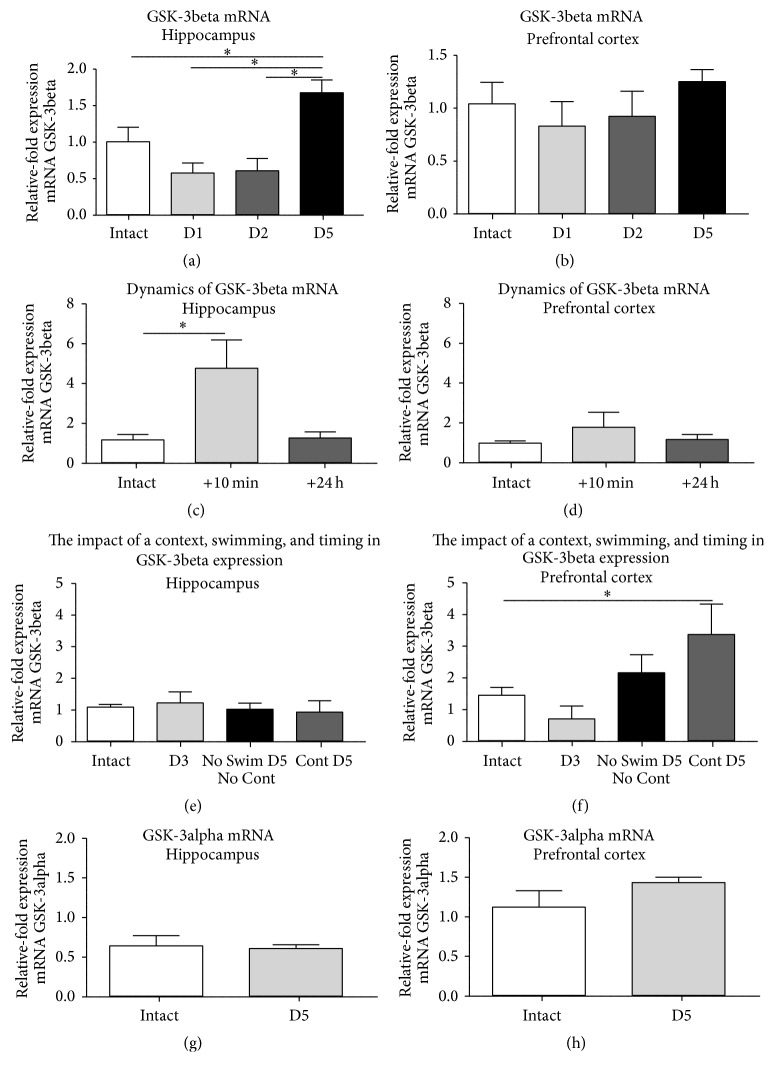
Brain expression of GSK-3beta in the modified swim test and the role of context, swimming, and timing of testing. (a) In comparison to intact controls (*n* = 11), hippocampal GSK-3beta mRNA levels were significantly elevated only on Day 5 (*n* = 14), but not on Day 1 (*n* = 12) or Day 2 (*n* = 11). (b) In the prefrontal cortex, the GSK-3beta mRNA levels were not changed on Day 1, Day 2, or Day 5; group sizes as indicated above. As compared with intact mice (n = 6), there was no change in GSK-3beta mRNA in mice sacrificed 24 h after swim session on Day 5 in the hippocampus (c), nor in the prefrontal cortex (d); *n* = 14 in each experimental group. (e) In comparison to intact controls (*n* = 10), hippocampal GSK-3beta mRNA levels were not significantly altered in mice tested and sacrificed on Day 3 (*n* = 6) nor in mice sacrificed on Day 5 without any behavioural manipulations (*n* = 6) or exposed to a context at testing on Day 5 (*n* = 6). (f) In comparison to intact controls, GSK-3beta mRNA levels were not changed in the prefrontal cortex of mice exposed to testing on Day 3, nor in mice sacrificed on Day 5 without any behavioural manipulations, and significantly elevated in animals exposed to a context of testing on Day 5; group sizes as indicated above. As compared with intact mice (*n* = 9), in mice exposed to the modified swim test (*n* = 13), there was no change in GSK-3alpha mRNA levels nor (g) in the hippocampus, nor (h) in the prefrontal cortex. ^*∗*^ *p* < 0.05, ANOVA and Tukey's multiple comparisons test; see the text; +10 min: mice sacrificed 10 min after swim session; +24 h: mice sacrificed 24 h after swim session; D3: mice exposed to the swim session on Day 3 instead of a swim on Day 5; Cont D5: mice exposed to a context of testing instead of a swim session on Day 5; no swim D5: mice were not exposed to a swim nor a context of swimming test prior a sacrifice. D1: Day 1; D2: Day 2; D5: Day 5. Data are mean ± SEM.

A comparison of GSK-3beta mRNA levels in the hippocampus of intact mice and groups of mice sacrificed on Day 1, Day 2, or on Day 5 (Figure 1(a); see Suppl. Table 3) revealed significant group differences (*F* = 9.77; *p* < 0.0001, ANOVA; Figure 3(a)). In comparison to intact control group, this measure was significantly elevated only on Day 5 of testing (*q* = 3.977; *p* = 0.0355, Tukey's test; Figure 3(a)), but not on Day 1 and on Day 2 (*q* = 2.465; *p* = 0.3154 and *q* = 2.238; *p* = 0.3988, resp.; Figure 3(a)). Mice subjected to modified swim test had significantly higher hippocampal expression of GSK-3beta than two latter groups (*q* = 0.1753; *p* = 0.0001 and *q* = 6.3464; *p* = 0.0003, resp.; Figure 3(a)).

ANOVA revealed no significant group differences in GSK-3beta mRNA levels in the prefrontal cortex (*F* = 0.7102; *p* = 0.5523, ANOVA; Figure 3(b)). In comparison to the intact control group, this parameter was not significantly altered on Day 1, Day 2, and Day 5 (*q* = 0.9227; *p* = 0.9140, *q* = 0.5839; *p* = 0.9758, and *q* = 0.9656; *p* = 0.9030, resp., Tukey's test; Figure 3(b)). Mice subjected to modified swim test had no differences in the expression of GSK-3beta in this brain structure as compared to the two latter groups (*q* = 1.881; *p* = 0.5505 and *q* = 1.657; *p* = 0.6484, resp.).

In a separate experiment, we compared GSK-3beta mRNA levels in the brain of mice from the modified forced swim test experiment that were sacrificed either 10 min or 24 h after the last swimming session. ANOVA revealed group differences in this measure in hippocampus but not in prefrontal cortex (*F* = 6.170; *p* = 0.0103 and *F* = 0.8936; *p* = 0.4276, resp., ANOVA; Figures 3(c) and 3(d)).* Post hoc* Tukey's test showed a significant increase in GSK-3beta mRNA levels in the hippocampus of mice sacrificed 10 min but not 24 h after test, as compared to intact group (*q* = 4.459; *p* = 0.0160 and *q* = 0.1155; *p* = 0.9963, resp.; Figure 3(c)). No group differences in GSK-3beta mRNA levels in the prefrontal cortex was found,* post hoc* analysis showed a lack of changes for either group tested in the swim test, in comparison with intact group (*q* = 1.821; *p* = 0.4209 and *q* = 0.4293; *p* = 0.9506, resp., Tukey test; Figure 3(d)). These data independently replicate the findings with changes of GSK-3beta expression during modified forced swim test presented herein and define selected time point +10 min after testing as the likely optimal time period for assessment of this measure in behavioural protocols.

A comparison of GSK-3beta mRNA levels of intact mice and of mice subjected to the context of testing instead of testing itself on Day 5 (Figure 1(d)), animals that were not exposed to either swimming or context and sacrificed on Day 5 (Figure 1(e)) and mice tested in “premature” last swim on Day 3 (Figure 1(f)), did not reveal significant group differences for the hippocampus (*F* = 0.2499; *p* = 0.8605, ANOVA; Figure 3(e)), but for the prefrontal cortex (*F* = 3.907; *p* = 0.0231, ANOVA; Figure 3(d)). As compared to the intact control, GSK-3beta mRNA was significantly elevated in prefrontal cortex of mice subjected to the modified swim test in which swimming Day 5 was replaced with their exposure to a context of testing (*q* = 3.945; *p* = 0.0497, Tukey's test; Figure 3(f)); no other group differences were found in this measure. There were no significant changes in the hippocampal GSK-3beta mRNA between these groups (*q* = 0.7750; *p* = 0.9461; Figure 3(e)). These data provide evidence for a role of timing and exposure to the context of testing in increased GSK-3beta expression during repeated exposure to a swim test.

To control for the specificity of changes in GSK-3beta expression in mice subjected to the modified swim test, the expression of brain GSK-3alpha mRNA was assessed on Day 5 in the same experiment (Figure 1(a)). We found no differences in this measure in both hippocampus and the prefrontal cortex between intact mice and mice subjected to the modified swim test (*p* = 0.7833 and *p* = 0.1852, resp., *t*-test; Figures 3(g) and 3(h)), suggesting a specificity of the above-described alternations of GSK-3beta mRNA levels in these brain structures.

### 3.4. Changes of Brain Total and Phosphorylated GSK-3beta during Modified Swim Test

**Figure 4:**
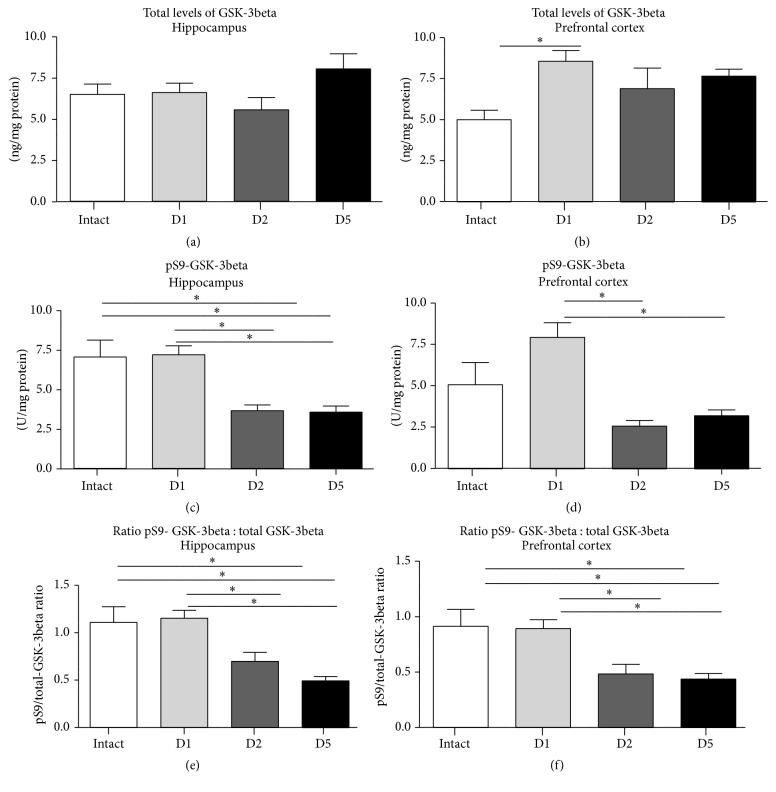
Brain levels of total and phosphorylated forms of GSK-3beta in the modified swim test. (a) In comparison to intact controls (*n* = 7), hippocampal level of total GSK-3beta was not significantly changed on Day 1 (*n* = 14), nor on Day 2 (*n* = 10) or on Day 5 (*n* = 12) of swim test. (b) In comparison to intact group, total level of GSK-3beta in the prefrontal cortex was significantly increased on Day 1 of swim test but not on Day 2 and Day 5; group sizes as indicated above. (c) In comparison to intact animals, hippocampal level of phosphorylated form of GSK-3beta was significantly decreased on Day 2 and Day 5, but not on Day 1 of the test; group sizes as indicated above. (d) In comparison to intact mice, the levels of pS9- GSK-3beta in prefrontal cortex were not altered on Day 2 and Day 5 of swim test; group sizes as indicated above. In comparison to intact controls, ratio of total/pS9 GSK-3beta (e) in the hippocampus and (f) in the prefrontal cortex was significantly decreased on Day 2 and Day 5, but not on Day 1; group sizes as indicated above. ^*∗*^ *p* < 0.05, ANOVA and Tukey's multiple comparisons test; see the text. D1: Day 1; D2: Day 2; D5: Day 5. Data are mean ± SEM.

ANOVA showed that there were no significant differences in brain total GSK-3beta in mice on Day 1, Day 2, and Day 5 of swim testing in the hippocampus, but in the prefrontal cortex (*F* = 1.958; *p* = 0.1363 and *F* = 3.016; *p* = 0.0420, ANOVA; Figures 4(a) and 4(b); see Supplementary Table 3). In the prefrontal cortex, brain total GSK-3beta was significantly higher on Day 1, but not on Day 2 and Day 5 of the swim test, where a nonsignificant increase of this measure was found in comparison with intact control (*q* = 4.144; *p* = 0.0283, *q* = 2.089; *p* = 0.4611, and *q* = 2.999; *p* = 0.1655, Tukey's test; Figures 4(a) and 4(b)).

There were significant group differences in the levels of pS9-GSK-3beta on Day 1, Day 2, and Day 5 of swim testing in the hippocampus and in the prefrontal cortex (*F* = 13.04; *p* < 0.0001 and *F* = 12.11; *p* < 0.0001, resp., ANOVA; Figures 4(c) and 4(d)).* Post hoc* analysis revealed a significant decrease of this measure in both of these brain areas only on Day 2 of swim test (*q* = 6.917; *p* = 0.0019 and *q* = 2.950; *p* < 0.0001, resp., Tukey's test; Figures 4(c) and 4(d)) and Day 5 (*q* = 6.618; *p* = 0.0002 and *q* = 6.868; *p* = 0.0001, resp.; Figures 4(c) and 4(d)), suggesting that repeated but not a single testing reduces the levels of inactive form of GSK-3beta.

Similarly, there were significant group differences in the ratio of levels of pGSK-3beta/total GSK-3beta between groups of mice sacrificed on Day 1, Day 2, and Day 5 of swim testing, both in the hippocampus and in the prefrontal cortex (*F* = 11.74; *p* < 0.0001 and *F* = 8.156; *p* = 0.0003, ANOVA; Figures 4(e) and 4(f)).* Post hoc* analysis revealed a significant decrease of this ratio in both of these brain areas on Day 2 (*q* = 5.111; *p* = 0.0048 and *q* = 4.449; *p* = 0.0172, Tukey's test; Figures 4(e) and 4(f)) and Day 5 (*q* = 7.410; *p* < 0.0001 and *q* = 5.662; *p* = 0.0017; Figures 4(e) and 4(f)). Moreover, the trend of the effects of a number of swim sessions on this parameter was found using univariate ANOVA for three groups and* post hoc* test for linear trend (hippocampus: slope −0.3301, *R* ^2^ = 0.5180; *p* < 0.0001 and prefrontal cortex: slope −0.2277, *R* ^2^ = 0.4006; *p* < 0.0001, Posttest for linear trend; Figures 4(e) and 4(f)).

### 3.5. Differential Activities of GSK-3beta in Mice with Low and High Floating in the Modified Swim Test

**Figure 5:**
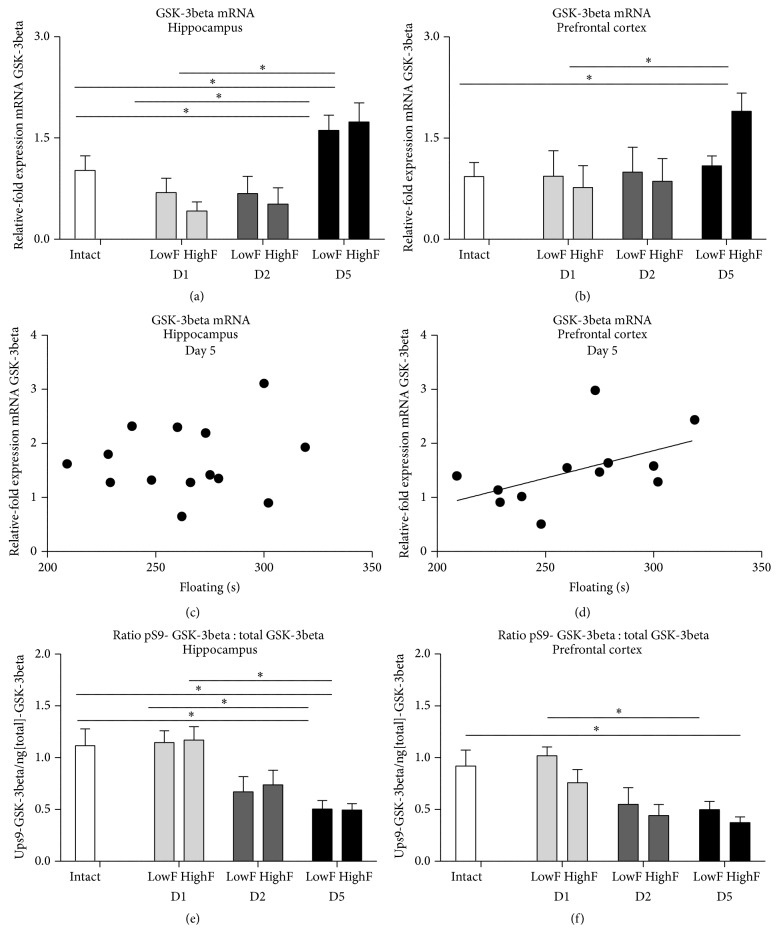
Brain activities of GSK-3beta in “high”- and “low-floaters” in the modified swim test. Mice were assigned to subgroups with “high-” (*n* = 7) or “low-” floaters (*n* = 7) using a mean of the groups as a criterion of a group formation. In comparison to intact controls, GSK-3beta mRNA (a) in the hippocampus and (b) in the prefrontal cortex was not significantly changed in either subgroup of mice on Day 1, Day 2, and Day 5 of swim test. (c) Hippocampal levels of GSK-3beta mRNA (*n* = 14) did not correlate with total duration of floating on Day 5 of swim test. (d) The levels of GSK-3beta mRNA in prefrontal cortex (*n* = 14) significantly correlated with the total duration of floating on Day 5 of a swim test. (e) In comparison to intact controls, hippocampal ratio of pS9 GSK-3beta /total GSK-3beta was significantly decreased both in “low” and “high” floating mice on Day 5, but not on Day 1 and Day 2. (f) In comparison to intact mice, the ratio of pS9 GSK-3beta /total GSK-3beta in prefrontal cortex was not significantly changed on Day 5 of swim test; size of groups as indicated above. ^*∗*^ *p* < 0.05, ANOVA and Tukey's multiple comparisons test; see the text; LowF: subgroup of mice classified as “low-floaters”; HighF: subgroup of mice classified as “high-floaters.” D1: Day 1; D2: Day 2; D5: Day 5. Data are mean ± SEM.

Mice were assigned to groups of “high-” and “low-floaters” based on the mean value of total duration of floating in the swim session preceding a sacrifice and compared for brain GSK-3beta activities (see Supplementary Table 4). Two-way ANOVA revealed no significant differences between these subgroups studied for GSK-3beta mRNA levels on Day 1, Day 2, and Day 5 both in the hippocampus and prefrontal cortex (*F* = 0.1973; *p* = 0.6588 and *F* = 0.3826; *p* = 0.5395, resp., two-way ANOVA; Figures 5(a) and 5(b)). Both high-floaters and low-floaters displayed significant increase of this measure in the hippocampus after swim session on Day 5 as compared to the session on Day 2 (*q* = 3.787; *p* = 0.0478 and *q* = 4.690; *p* = 0.0090, resp., Figure 5(a)); no significant differences between subgroups at this time point were found (*q* = 0.5363, *p* > 0.999).

As for the prefrontal cortex, “high-floaters” but not “low-floaters” showed significant increase of brain GSK-3beta mRNA on Day 5, as compared to session on Day 2 (“high-floaters”: *q* = 3.793; *p* = 0.0489; “low-floaters” *q* = 0.3287; *p* = 0.9955, resp., Tukey's test, Figure 5(b)). No significant differences between the subgroups were revealed in this measure (*q* = 0.4696; *p* > 0.9999, *q* = 0.4881; *p* > 0.9999, and *q* = 2.856; *p* = 0.1695; Figure 5(b)). No other significant group differences were found.

No significant correlation was found between GSK-3beta mRNA levels in the hippocampus and the duration of floating behaviour on Day 1, Day 2, or Day 3 (*r* = −0.06993; *p* = 0.8346, *r* = −0.2545; *p* = 0.4511, and *r* = 0.008801; *p* = 0.9790, resp., Spearman; Figure 5(c)). Spearman correlation analysis showed a significant correlation between mRNA levels of GSK-3beta in the prefrontal cortex and the duration of floating on Day 5 (*r* = 0.5944; *p* = 0.0457, Spearman) but not on Day 1 nor on Day 2 (*r* = −0.9524; *p* = 0.8401 and *r* = 0.1405; *p* = 0.6449, Spearman; Figure 5(d)).

Two-way ANOVA showed no significant differences in ratio pS9-GSK-3beta/total GSK-3 in the hippocampus on Day 1, Day 2, and Day 5 (*F* = 0.04540; *p* = 0.8324, two-way ANOVA; Figure 5(e)). At testing session on Day 5, as compared to session on Day 1, this measure was significantly decreased in the hippocampus (*q* = 4.209; *p* = 0.0247 and *q* = 5.136; *p* = 0.0043, resp., Tukey's test; ANOVA; Figure 5(e)) and in the prefrontal cortex (*q* = 4.389; *p* = 0.0184 and *q* = 2.990; *p* = 0.1675; Figure 5(f)) of both “high-” and “low-floaters.” No significant differences between subgroups of “high-” and “low-floaters” were revealed at all time points in the hippocampus (*q* = 0.1931; *p* > 0.9999, *q* = 0.4485; *p* > 0.9999, and *q* = 0.05110; *p* > 0.9999, resp.; Figure 5(e)) nor in the prefrontal cortex (*q* = 2.194; *p* = 0.7747, *q* = 0.6684; *p* = 0.9997, and *q* = 0.9733; *p* = 0.9968, resp.; Figure 5(f)). No other significant group differences were found.

### 3.6. The Effects of Imipramine on Floating Behaviour and GSK-3beta Function in the Modified Swim Test

**Figure 6:**
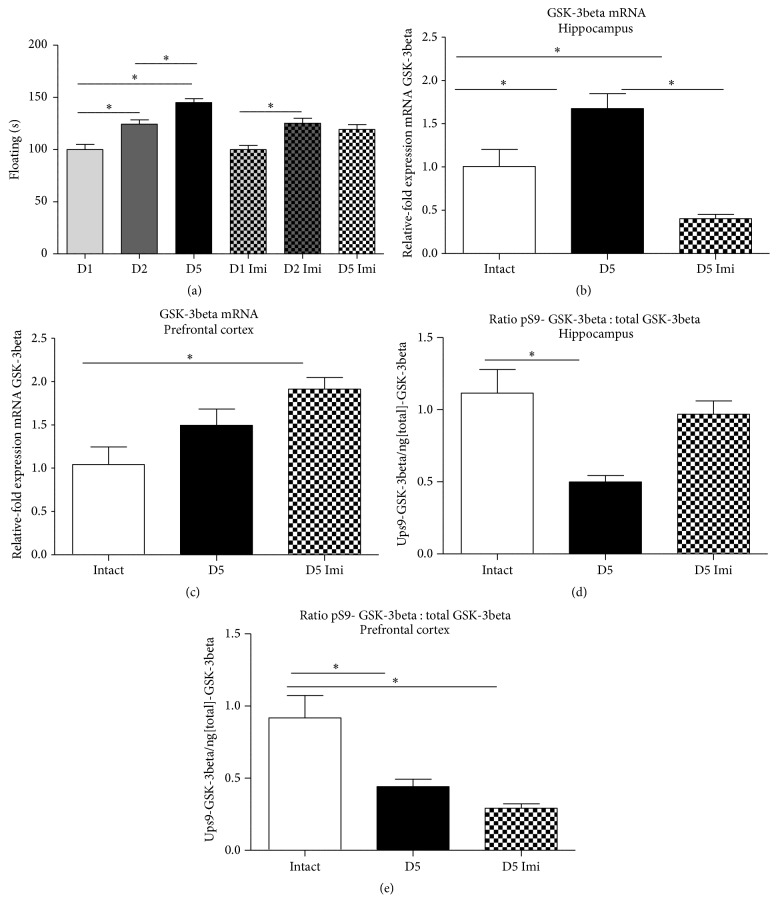
The effects of imipramine on floating behaviour and brain GSK-3beta activities in the modified swim test. (a) Imipramine-treated mice (*n* = 14) showed no significant increase of floating behaviour as compared with control group (*n* = 14), from Day 2 to Day 5. As compared to intact controls (*n* = 13), on Day 5, GSK-3beta mRNA of imipramine-treated mice was (b) decreased in the hippocampus and (с) not changed in the prefrontal cortex; group sizes as indicated above. (d) As compared to intact mice, imipramine-treated group had significantly increased ratio of total/pS9 of GSK-3beta in the hippocampus on Day 5; group sizes as indicated above. (e) As compared with intact mice, there were no significant changes the in ratio of pS9 GSK-3beta /total GSK-3beta in the prefrontal cortex of imipramine-treated mice on Day 5 of swim test; group sizes as indicated above. ^*∗*^ *p* < 0.05, ANOVA and Tukey's multiple comparisons test; see the text; Imi: imipramine-treated group; D1: Day 1; D2: Day 2; D5: Day 5. Data are mean ± SEM.

ANOVA repeated measurements test showed that while nontreated control groups displayed a significant increase in floating behaviour from Day 2 to Day 5, no such increase was found in mice dosed with a low dose of antidepressant imipramine (*q* = 5.386; *p* = 0.0038 and *q* = 2.058; *p* = 0.3412, resp., Tukey's test; Figure 6(a)) (see Supplementary Table 5). Of note, there was no difference between control and imipramine-treated mice on Day 1 (*q* = 1.017*e* − 006; *p* > 0.9999; Figure 6(a)) and on Day 2 (*q* = 1.163; *p* > 0.9999; Figure 6(a)), suggesting that applied dose was too low to be effective in the classic protocols of Porsolt's test. GSK-3beta mRNA levels were significantly altered in the hippocampus and were not changed in the prefrontal cortex and between the groups (*F* = 20.05; *p* < 0.0001 and *F* = 6.164; *p* = 0.0053, resp., ANOVA: Figures 6(b) and 6(c)). Tukey's* post hoc* test showed a significant decrease of this parameter in the hippocampus of imipramine-treated mice, as compared with pharmacologically naïve group, on Day 5 (*q* = 8.952; *p* < 0.0001; Figure 6(b)). There was no such difference found for the prefrontal cortex (*q* = 2.584; *p* = 0.1765; Figure 6(c)). A ratio pS9-GSK-3beta/total GSK-3beta among the groups was significantly altered in the hippocampus and in the prefrontal cortex (*F* = 10.65; *p* = 0.0005 and *F* = 13.73; *p* = 0.0001, resp., ANOVA; Figures 6(d) and 6(e)).* Post hoc* analysis showed a significant reduction of this measure in nontreated mice as compared to intact animals (*q* = 5.989; *p* = 0.0008, Tukey's test; Figure 6(d)), while no such difference was found between intact mice and imipramine-treated group (*q* = 1.425, *p* = 0.5792; Figure 6(d)), suggesting that antidepressant treatment preserved normal levels of ratio pS9-GSK-3beta/total GSK-3beta in this brain structure. In the prefrontal cortex, a ratio pS9-GSK-3beta/total GSK-3beta was decreased in both groups subjected to modified forced swim test, as compared to intact control (*q* = 5.779; *p* = 0.0013 and *q* = 7.108; *p* = 0.0001; Figure 6(e)); there was no difference between the latter groups (*q* = 1.904; *p* = 0.3849; Figure 6(e)).

## 4. Discussion

While repeated exposure to adversities including swimming under inescapable conditions has previously been used to achieve more robust depressive phenotypes by increasing the intensity of stress [1, 20, 21], the protocol of repeated swimming proposed here was designed on a different basis. We have hypothesized that floating behaviour in additional swimming session of Porsolt's test in the mouse that is postponed from the initial noxious experience of swimming might be a better predictor of individual depressive-like behavioural and molecular changes than when measured in the original commonly used protocols of this model, as it likely implements an component of cognitive processing of adverse experiences. Present results support this assumption showing that similar factors to those that are crucial for acquisition of contextual memories in experimental models of learning, such as reexposure to the context of testing, timing between challenge, and development of behavioural response [52, 53], are also important for the depressive-like behavioural and molecular effects in the repeated swim test with delayed testing. Moreover, modification of the swim test applied here has enabled us to differentiate the individual animal's susceptibility to a depressive-like state that so far was not achieved with the original Porsolt test. Together, our data provide evidence for a higher accuracy of the modified swim test in modeling depressive states and further support the hypothesis that enhanced contextual learning and consolidation of contextual memories impact the development of a depressive-like response.

Despite consideration that during delayed swim session, stress-related recall of an initial adverse experience may be extinguished and consequently animals display decreased floating behaviour, we found an opposite effect. Floating behaviour was further increased during the postponed swimming session and was prevented by antidepressant treatment of mice with a low dose of imipramine. Importantly, this potentiation of floating behaviour was evident in the very first minutes of testing and did not develop during the course of additional stressful swimming experience, suggesting that the animals were prone to floating already by the initial exposure to the additional swim test. The data reported here are in line with previously obtained findings, which showed that memory attenuation by various compounds, including NMDA-receptor and protein synthesis blockers, as wells as acetylcholinesterase inhibitors, may reduce floating behaviour, thus suggesting a role of contextual conditioning during repeated forced swimming [13, 54, 55]. Floating in the Porsolt test might therefore be called “learned immobility” and considered as an adaptive process that has been discussed in the literature since the introduction of this paradigm [56]. In this light, changes in GSK-3beta activities in the hippocampus and prefrontal cortex during forced swimming could provide further input in the interpretation of a physiological significance of floating behaviour in in the mouse during this procedure.

The potentiation of behavioural despair on Day 5 was accompanied by an upregulation of GSK-3beta, both a mediator of synaptic plasticity processes and a marker of a distress and depressive state in the brain [21, 25, 28, 30]. The lack of changes in GSK-3alpha mRNA suggests specificity of the observed enhanced expression of GSK-3beta. While analysis across experimental groups revealed elevated expression of GSK-3beta in the hippocampus, but not in the prefrontal cortex, a differentiation of mice by floating behaviour into subgroups according to their scores of depressive-like behaviour showed that animals defined as “high-floaters” displayed upregulated GSK-3beta in the prefrontal cortex as compared to “low-floaters” with a correlation between the duration of floating at Day 5 and GSK-3beta expression. Interestingly, there was no difference in GSK-3beta expression in the hippocampus between “high-floaters” and “low-floaters” as well as no correlation between hippocampal GSK-3beta mRNA and floating behaviour. Our results suggest that both hippocampus and prefrontal cortex are implicated in the formation of a depressive-like state in mice exposed to the modified swim test, but circuitry's function or/and dynamics of involvement in these processes are likely to be different. This is in line with other studies that suggest differential roles of these limbic structures in the mechanisms of behavioural despair, fear memories, and learned helplessness. Studies in recent years dissected the roles of particular regions of prefrontal cortex in the control of activity of amygdala during fear learning and its dependence on input from the hippocampus at early stages of consolidation, while distinct areas of the hippocampal formation were shown to be involved in long-term storage of contextual memory and stress response [35, 57–62]. Based on this evidence, similar GSK-3beta expression of “high-floaters” and “low-floaters” in the hippocampus may be interpreted as a molecular signature of a similar context recall in two groups that is not related to the development of a “depressive-like state.” In contrary, given above-discussed role of the prefrontal cortex and GSK-3beta in the mechanism of stress and depression, elevated expression of this gene in the prefrontal cortex exclusively in a “high-floaters” group might be regarded as a molecular correlate of a depressive-like state in mice which are predisposed to this condition.

Reported behavioural and molecular changes were found on Day 5, but not in the testing protocols of the original Porsolt paradigm. Furthermore, they were largely prevented by a low dose of imipramine, which did not alter floating behaviour in the classic variants of the swim test, but in our modified protocol with a delayed session. Our finding suggests specific sensitivity of the neurobiological processes preceding the delayed swim test and their relevance for the pathophysiological mechanism of depressive-like states. Moreover, this pharmacologically validates our modified swim test as a model of a depressive-like state in rodents and suggests its improved sensitivity to antidepressant effectiveness of novel compound compared to commonly employed protocols of Porsolt's test.

Finally, administration of imipramine precluded changes in the GSK-3beta both at the gene and protein level in the hippocampus, but not in the prefrontal cortex, further underscoring a differential role of these brain structures in the pathophysiology of depressive-like behaviour of helplessness and despair associated with this paradigm. Evidence suggests differential dynamics of gene and protein expression of GSK-3beta as well as compensatory changes in interrelated structures of the limbic system, under various conditions related to stress and learning of adverse memories [25, 26, 63] which are likely to explain distinct changes in brain GSK-3beta of imipramine-treated mice.

Increased GSK-3beta mRNA expression in the hippocampus and prefrontal cortex of mice subjected to repeated swimming was accompanied by a substantial reduction of an inactive form of GSK-3beta, its phosphorylated form, and the pGSK-3beta/GSK-3beta ratio, as well-established molecular correlates of distress and depressive conditions [64, 65]. The profound decrease of this ratio in both the hippocampus and prefrontal cortex further emphasizes the role of repeated swimming in the development of depressive-like behavioural and molecular changes observed in current study. Besides, while phosphorylation of GSK-3beta at 9-serine is considered to be the main regulatory mechanism of GSK-3beta function, based on the recent literature [66, 67], it might be hypothesized that an increase of GSK-3beta activities via phosphorylation at Tyr216 may occur under current experimental conditions and interfere with changes of this kinase at the gene and protein level reported here. This could be addressed in the next experiments with the modified swim test.

The pGSK-3beta/GSK-3beta ratio was similar on Day 2 and Day 5 of testing and no differences in these measures were found between “high-” and “low-” floaters, possibly reflecting limited sensitivity of this approach. As discussed above, the discrepancy between gene and protein parameters of GSK-3beta function on Day 2 and in the effects of imipramine on the prefrontal cortex might also be due to distinct dynamics of changes of gene expression and GSK-3beta phosphorylation and compensatory effects. We recently showed that while mice sacrificed 24 h after swim in the modified swimming test display no changes in GSK-3beta at the gene expression level, they have elevated GSK-3beta protein levels, suggesting that gene and protein changes in GSK-3beta may not always occur in parallel (Shevtsova and Markova, unpublished data). Generally, these results demonstrate that repeated but not single exposures to a swim test may result in an increase of activity of brain GSK-3beta at the protein level. Rapid changes of GSK-3beta activities on the gene and protein levels were previously reported; they were found as early as less than 10 min after challenge, while frequently fading away 24 h thereafter [26, 63, 66].

A series of experiments that addressed the role of reexposure of mice to a context of testing and timing in our modified swim test revealed their pivotal importance for the occurrence of behavioural and molecular changes observed in this paradigm. Study with a replacement of a swim session with an exposure to the context of testing on Day 2 and Day 5 has shown similar increases in floating and GSK-3beta gene expression to those observed in a conventional protocol of this model. These data, together with the finding that the omission of intermediate swimming or context exposure on Day 2 prevented an increase in floating, demonstrate crucial role of contextual components in both behavioural and molecular depressive-like features, which are induced in our swim model. A “premature” exposure to swimming and analysis of GSK-3beta changes on Day 3 showed a lack of changes that were typical for a conventional swim testing with a sacrifice on Day 5, thus pointing to the critical role of timing in the development of depressive-like behavioural and molecular features. These findings indicate that analogous to the processes of consolidation of learning experiences in mouse models of contextual memory [52, 53], the formation of a depressive-like phenotype requires a “consolidation phase.” However, this hypothesis remains to be further explored assessing additional markers of molecular, structural, and functional plasticity. Such studies are in progress.

In summary, given the well-known role of GSK-3beta in neuronal and synaptic plasticity related to learning and memory on one hand and its importance as a factor of distress and maladaptation on the other hand, it may be speculated that GSK-3beta mediate pathologically enhanced consolidation of memories associated with adverse experience resulting in a depressive-like phenotype. Further studies are required to elucidate the role and the mechanism of enhanced learning in the context of adversity as the neurobiological basis of individual predisposition to depressive illness.

## 5. Conclusions

Taken together, the modified paradigm we propose offers a practical approach to study the role of enhanced cognitive processing in the pathophysiological mechanisms of depression and allows dissect interindividual differences in the susceptibility to this condition in mice. While classical versions of Porsolt's test, commonly regarded as tests for a depressive-like behaviour, do not* per se* mimic this condition, modified swim procedure does evoke the neurobiological changes that are relevant to a depressive-like state as proposed here. Hence, the modified swim test can be comparable to chronic depression paradigms, for example, chronic stress, learned helplessness, and similar models, which possess substantial construct validity and etiological relevance that cannot be attributed to short behavioural tests for a depressive-like behaviour, like “classic” Porsolt's and tail suspension tests. Importantly, proposed model, for the first time, may enable a tracking of an exact temporal relationship between an event that induces a depressive-like state and a development its molecular/neurobiological trace in dynamics that in such sense is virtually impossible with chronic models of depression. Given this and also minimal experimental work requirement of the new model proposed here, we believe that the modified swim test may provide a methodological solution for combining the advantages of chronic models of depression, while overcoming their disadvantages of highly demanding resources they typically require, as well as improving laboratory animal welfare.

## Supplementary Material

Supplementary data file contains experimental details on RT PCR and sequences of primers used (Table 1), as well as a summary of the experiments that illustrate which groups and which measures were compared in present work, alone with the outcomes from these comparisons. Tables 2 and 3 sum up the schemes of the experiments that were done to study the roles of swimming, context and timing of testing as factors of floating behaviour and GSK3-beta mRNA in the modified swim test; their outcome presented also in Figs. 2 and 3. Table 4 summarizes data comparisons in floating and GSK3-beta activities between Day 1-, Day 2- and Day 5- (modified) swim test protocols, also in “low-floaters” vs “high-floaters” (data are presented in Figs.4 and 5). Table 5 presents studies and the results concerning effects of imipramine on floating and GSK3-beta activities in the modified forced swim test (data are presented in Fig.6).
