# Supplementary material for: Individual Differences in Behavioural Despair Predict Brain GSK-3beta Expression in Mice: The Power of a Modified Swim Test
Source: Neural Plast. 2016 Jul 5;2016:5098591. doi: 10.1155/2016/5098591 (PMC4949347; doi:10.1155/2016/5098591)
Supplement: Supplementary file 1 — Supplementary data file contains experimental details on RT PCR and sequences of primers used (Table 1), as well as a summary of the experiments that illustrate which groups and which measures were compared in present work, alone with the outcomes from these comparisons. Tables 2 and 3 sum up the schemes of the experiments that were done to study the roles of swimming, context and timing of testing as factors of floating behaviour and GSK3-beta mRNA in the modified swim test; their outcome presented also in Figs. 2 and 3. Table 4 summarizes data comparisons in floating and GSK3-beta activities between Day 1-, Day 2- and Day 5- (modified) swim test protocols, also in “low-floaters” vs “high-floaters” (data are presented in Figs.4 and 5). Table 5 presents studies and the results concerning effects of imipramine on floating and GSK3-beta activities in the modified forced swim test (data are presented in Fig.6). [file 5098591.f1.pdf]

Supplementary data

***Table 1*. SEQUENCES OF PRIMERS USED**

**Gene**	**Forward primer 5′–3′**	**Reverse primer 5′–3′**
GAPDH	TGCACCACCAACTGCTTAG	GGATGCAGGGATGATGTTC
GSK-3 alpha	AATCTTGGCCAGTCTGAGCT	TCAGTCCTGGTGAACTGTCC
GSK-3 beta	TCCATTCCTTTGGAATCTGC	CAATTCAGCCAACACACAGC

RNA isolation and RT PCR

The qRT-PCR *was performed in a 25 µl reaction volume containing a 10×PCR Buffer (2.5µl), 25 mmol MgCl2 (4 µl), 10 mmol dNTPs (2µl), specific forward and reverse primers at 20 pmol/µl concentration (1 µl), cDNA (2µl), 5 u/µl Taq DNA polymerase (1 µl) (Beagle, st. Petersburg, Russia), and ddH2O (10 µl). All samples were run in duplicate. Cycling was performed at 95 C° for 5 min followed by a 45-cycle amplification at 95°C for 10s, then at the annealing temperature defined previosuly for 15 s and at the temperature 72°C for 20 s.*

Results of the qPCR measurements were expressed as Ct values, where Ct is defined as the threshold cycle of PCR at which amplified product was 0.05 % of normalized maximal signal. We used the comparative Ct method and computed the difference between the expression of the gene of interest and GAPDH expression in each cDNA sample (2−ΔΔCt method). Results are expressed as folds of expression compared to the mean values of expression in non-stressed control animals (Couch et al., adapted from Livak and Schmittgen 2001).

Table 2. **FLOATING IN THE MODIFIED SWIM TEST: ROLES OF SWIMMING, CONTEXT AND TIMING (Fig. 2)**

FACTOR / EXPERIMENT	Swimming vs context	Role of context	Role of timing
**Protocol**	Fig. 1 B swimming is replaced by a context exposure on Day 2	Fig. 1 C no swimming, no context exposure on Day 2	Fig. 1 F swimming is on Day 3 instead of Day 5
**Outcome: floating on Day 5**	↑	=	=

Table 2. Floating in the modified swim test: roles of swimming, context and timing. *In series of experiments, the roles of swimming experience vs. context exposure, a reminder of a context of swimming, and a factor of timing of testing, in an increase of floating behaviour on Day 5 of the modified swim test, were dissected. Mice were exposed to the modified swim test with additional delayed testing on Day 5 or several variants of this protocol presented on Fig. 1. In comparison to the preceding session, the duration of floating behaviour on Day 5 was augmented (↑) or not altered (=) in applied protocols of testing.*

Table 2. **BRAIN mRNA GSK3 BETA IN MODIFIED SWIM TEST: ROLES OF SWIMMING, CONTEXT AND TIMING (Fig. 3)**

FACTOR / EXPERIMENT	Swimming vs context	Role of context	Role of timing
**Protocol**	Fig. 1 D; swimming is replaced by context exposure on Day 5	Fig. 1 E; no swimming, no context exposure on Day 5	Fig. 1 F; swimming on Day 3 instead Day 5
**Outcome: mRNA GSK3 beta**	Hip =<br>PFC ↑	=	=

Table 3. mRNA GSK3 beta in the modified swim test: roles of swimming, context and timing. *In series of experiments, the roles of swimming experience vs. context exposure, a reminder of a context of swimming, and a factor of timing of testing, in an increase of mRNA GSK3 beta on Day 5 of the modified swim test, were addressed. Mice were exposed to the modified swim test with additional delayed testing on Day 5 or several variants of this protocol presented on Fig. 1. In comparison to the preceding session, the mRNA GSK3 beta level in the hippocampus (Hip) and prefrontal cortex (PFC) on Day 5 was augmented (↑) or not altered (=) in applied protocols of testing.*

Table 4. **A COMPARISON OF FLOATING AND GSK3 BETA BRAIN ACTIVITES BETWEEN DAY 1-, DAY 2- AND DAY 5- (MODIFIED) SWIM TEST PROTOCOLS**

PARAMETER / TEST SESSION	FLOATING	mRNA GSK3 beta		pGSK3 beta / Total GSK3 beta	
		**Hip**	**PFC**	**Hip**	**PFC**
**Day 1**		vs Int =	vs Int =	vs Int =	vs Int =
**Day 2**	vs Day 1 ↑	vs Int =<br>vs Day 1 =	vs Int =<br>vs Day 1 =	vs Int ↓<br>vs Day 1 ↓	vs Int ↓<br>vs Day 1 ↓
**Day 5**	vs Day 1 ↑<br>vs Day 2 ↑	vs Int LF ↑,<br>HF ↑<br>vs Day 1 LF ↑,<br>HF ↑	vs Int LF =,<br>HF ↑<br>vs Day 1 LF = ,<br>HF ↑	vs Int LF ↓,<br>HF ↓<br>vs Day 1 LF ↓,<br>HF ↓	vs Int LF ↓<br>vs Day1 HF ↓

Table 4. A comparison of floating and GSK3 beta brain activities between Day 1-, Day 2- and Day 5- (modified) swim test protocols. Total duration of floating, brain *GSK3 beta gene expression and protein levels* were *compared between the groups of mice sacrificed 10 min after testing at Day 1, Day 2 or delayed session on Day 5, in the swim test. mRNA GSK3beta and a* ratio pGSK3 beta / Total GSK3 beta were evaluated in the hippocampus *(Hip)* and prefrontal cortex *(PFC)* of subgroups of mice defined as Low Floaters (**LF**, see manuscript text) and High Floaters (**HF**, see manuscript text) post-testing on Day 2 and Day 5, and were compared to the values determined in intact mice (**Int,** see

manuscript text) and in mice sacrificed at Day 1 of testing. A scheme of testing / sacrifice for each *protocol is presented on Fig. 1A. Evaluated behavioural and molecular measures were augmented (↑),* decreased **(↓**) *or not altered (*=*) in above-described comparisons.*

Table 5. **EFFECTS OF IMIPRAMINE ON FLOATING AND GSK3 BETA ACTIVITIES IN THE MODIFIED FORCED SWIM TEST**

PARAMETER / TREATMENT	FLOATING	mRNA GSK3 beta		pGSK3 beta / Total GSK3 beta	
		**Hip**	**PFC**	**Hip**	**PFC**
**Day 5 No Drug**	vs Day 1 ↑<br>vs Day 2 ↑	vs Int ↑<br>vs Day 1 ↑<br>vs Day 2 ↑	vs Int =	vs Int ↓	vs Int ↓
**Day 5 Imi**	vs Day 1 ↑<br>vs Day 2 =	vs Int ↓<br>vs Con (No drug) ↓	vs Int ↑<br>vs Con (No drug) =	vs Int =<br>vs Con (No drug)↑	vs Int ↓<br>vs Con (No drug) =

Table 5. Effects of imipramine on floating and GSK3 beta activities in the modified forced swim test. Total duration of floating, brain *GSK3 beta gene expression and protein levels* were *compared between the groups of mice sacrificed 10 min after testing at delayed session on Day 5 of the swim test, which either received no drug or were treated with a low dose of imipramine. mRNA GSK3beta and a* ratio pGSK3 beta / Total GSK3 beta were evaluated in the hippocampus *(Hip)* and prefrontal cortex *(PFC)* of these groups and were compared to the values determined in intact mice (**Int,** see manuscript text) and in animals sacrificed at Day 1 and Day 2 of testing. A scheme of testing / sacrifice for each *protocol is presented on Fig. 1G. Evaluated behavioural and molecular measures were augmented (↑),* decreased **(↓**) *or not altered (*=*) in above-described comparisons.*
