# Supplementary material for: Individual Differences in Behavioural Despair Predict Brain GSK-3beta Expression in Mice: The Power of a Modified Swim Test
Source: Neural Plast. 2016 Jul 5;2016:5098591. doi: 10.1155/2016/5098591 (PMC4949347; doi:10.1155/2016/5098591)
Supplement: Supplementary file 2 [file 5098591.f2.pdf]

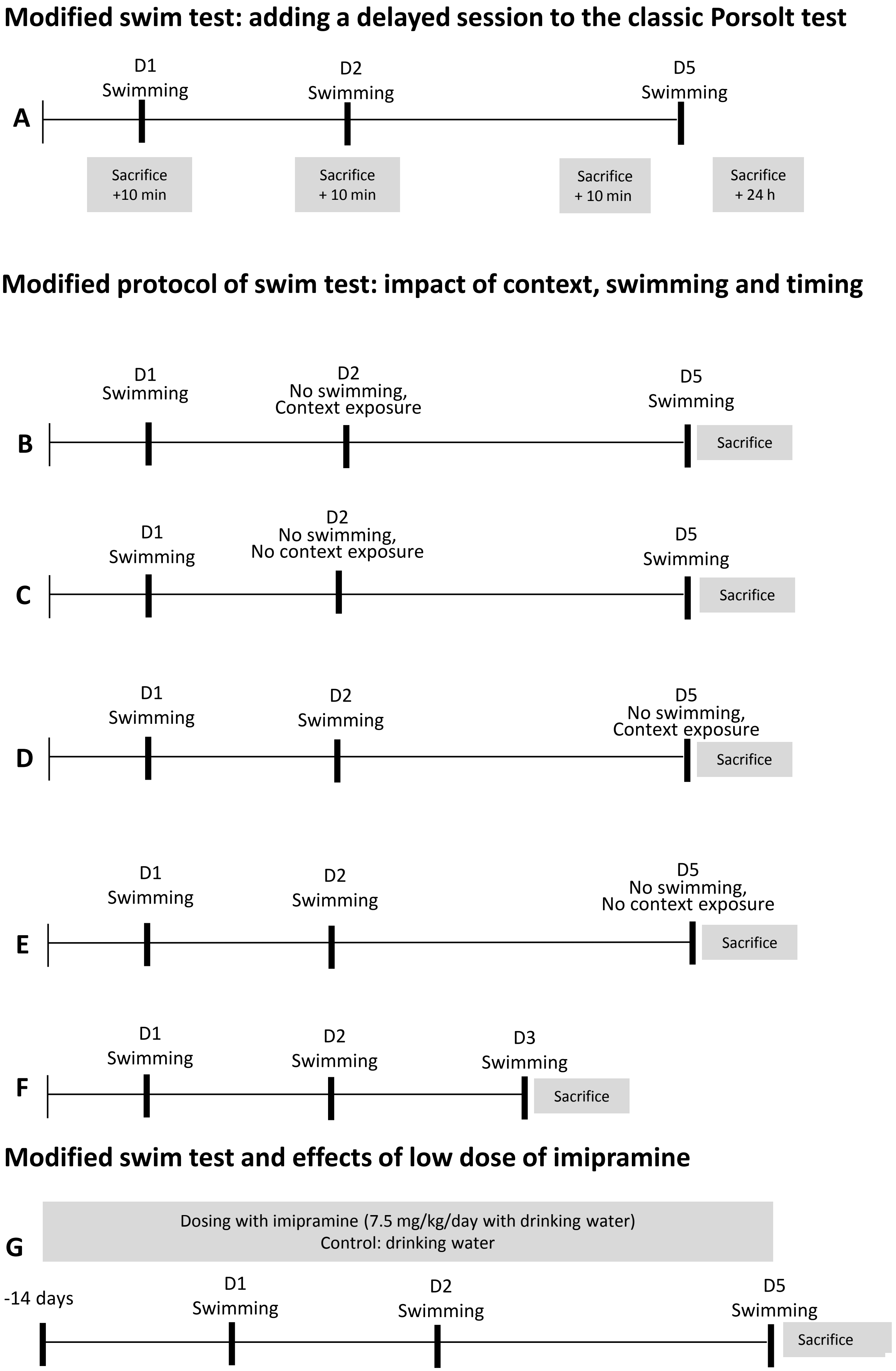
Modified swim test: adding a delayed session to the classic Porsolt test
A
D1 Swimming
D2 Swimming
D5 Swimming
Sacrifice +10 min
Sacrifice + 10 min
Sacrifice + 10 min
Sacrifice + 24 h
Modified protocol of swim test: impact of context, swimming and timing
B
D1 Swimming
D2 No swimming, Context exposure
D5 Swimming
Sacrifice
C
D1 Swimming
D2 No swimming, No context exposure
D5 Swimming
Sacrifice
D
D1 Swimming
D2 Swimming
D5 No swimming, Context exposure
Sacrifice
E
D1 Swimming
D2 Swimming
D5 No swimming, No context exposure
Sacrifice
F
D1 Swimming
D2 Swimming
D3 Swimming
Sacrifice
Modified swim test and effects of low dose of imipramine
G
Dosing with imipramine (7.5 mg/kg/day with drinking water)
Control: drinking water
-14 days
D1 Swimming
D2 Swimming
D5 Swimming
Sacrifice

Figure 1

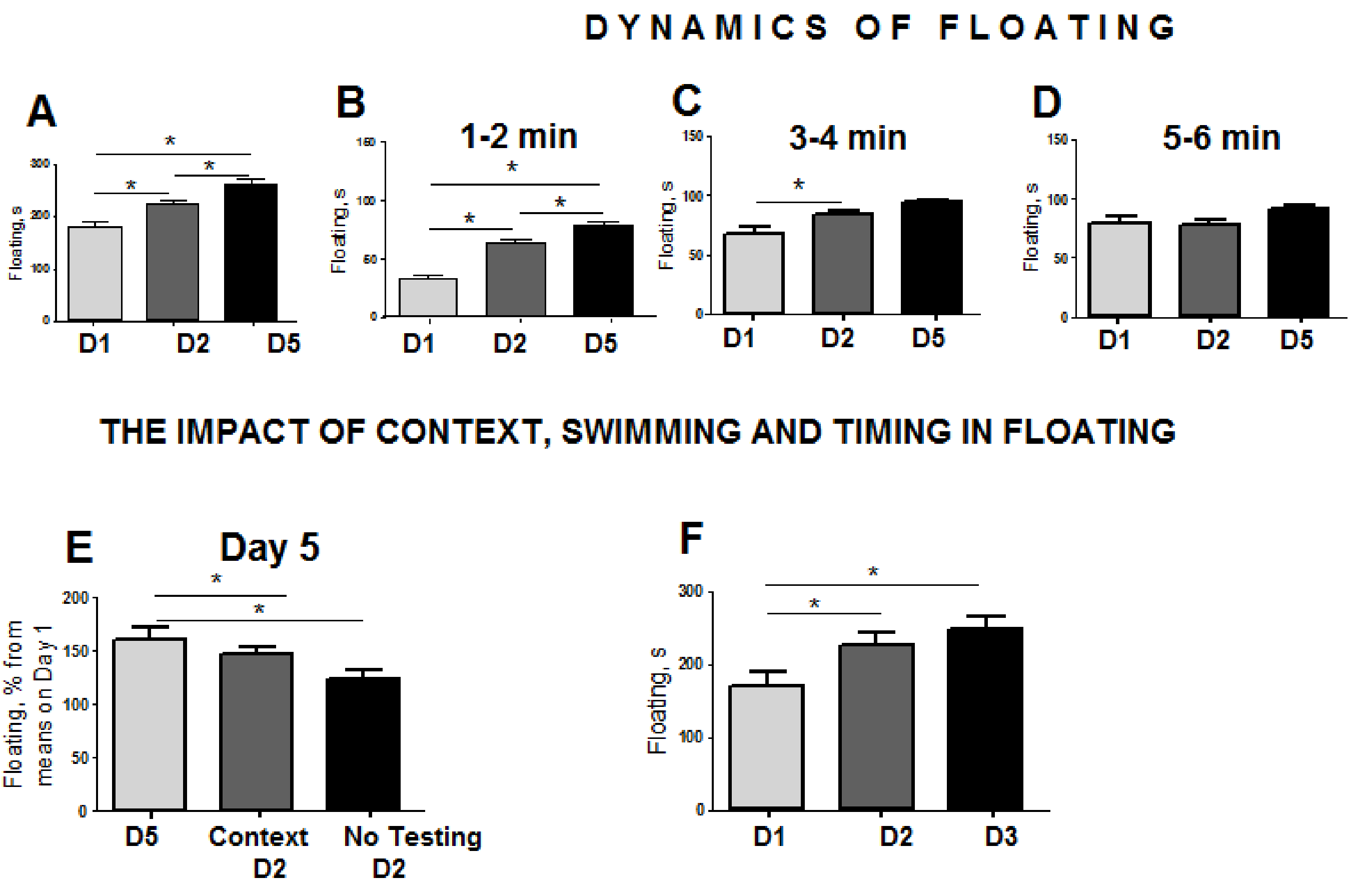
DYNAMICS OF FLOATING
A
Floating, s
D1
D2
D5
B
1-2 min
Floating, s
D1
D2
D5
C
3-4 min
Floating, s
D1
D2
D5
D
5-6 min
Floating, s
D1
D2
D5
THE IMPACT OF CONTEXT, SWIMMING AND TIMING IN FLOATING
E
Day 5
Floating, % from means on Day 1
D5
Context D2
No Testing D2
F
Floating, s
D1
D2
D3

Figure 2

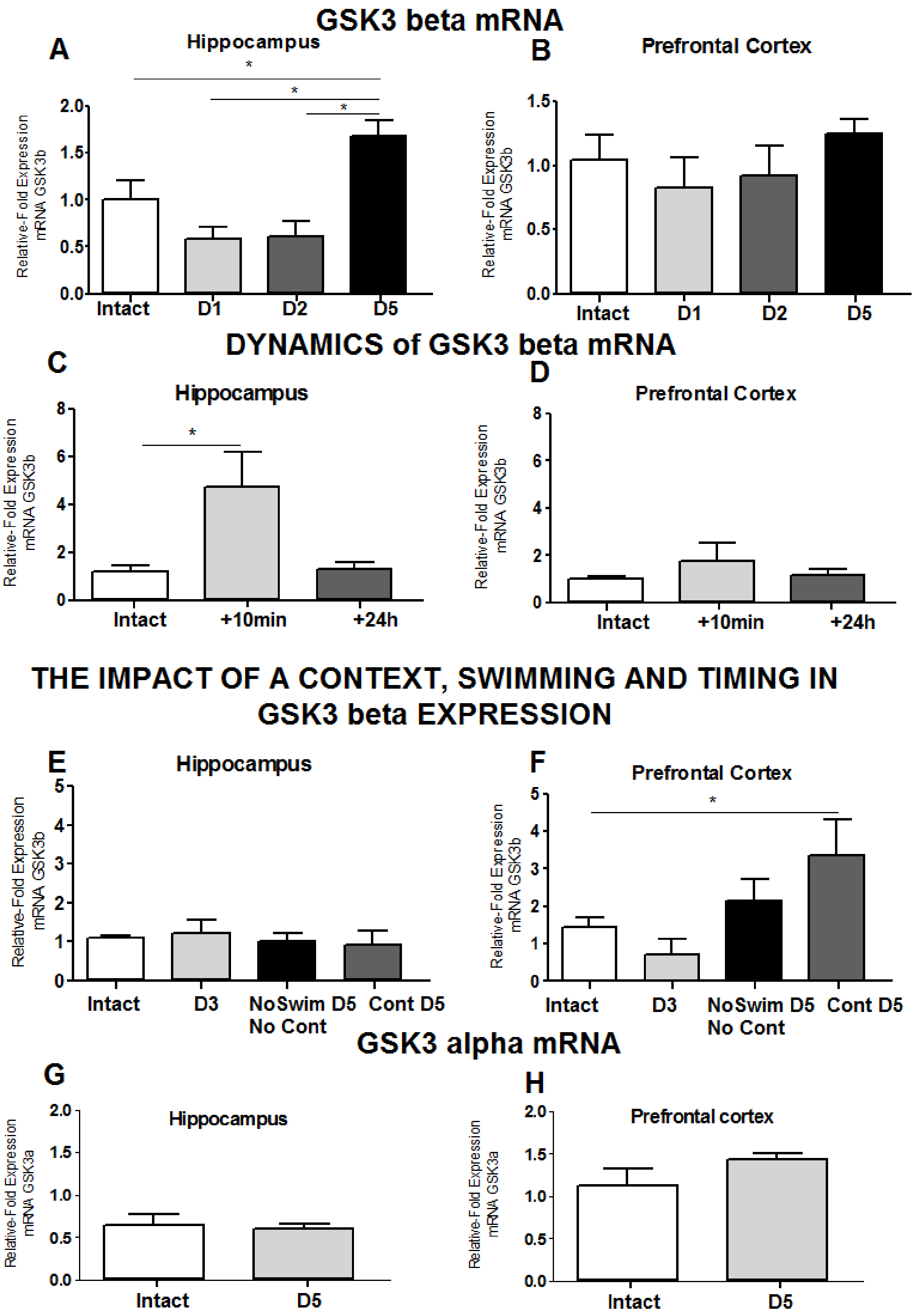
GSK3 beta mRNA
A
Hippocampus
B
Prefrontal Cortex
Relative-Fold Expression mRNA GSK3b
Intact
D1
D2
D5
DYNAMICS of GSK3 beta mRNA
C
Hippocampus
D
Prefrontal Cortex
Relative-Fold Expression mRNA GSK3b
Intact
+10min
+24h
THE IMPACT OF A CONTEXT, SWIMMING AND TIMING IN GSK3 beta EXPRESSION
E
Hippocampus
F
Prefrontal Cortex
Relative-Fold Expression mRNA GSK3b
Intact
D3
NoSwim D5 No Cont
Cont D5
GSK3 alpha mRNA
G
Hippocampus
H
Prefrontal cortex
Relative-Fold Expression mRNA GSK3a
Intact
D5

Figure 3

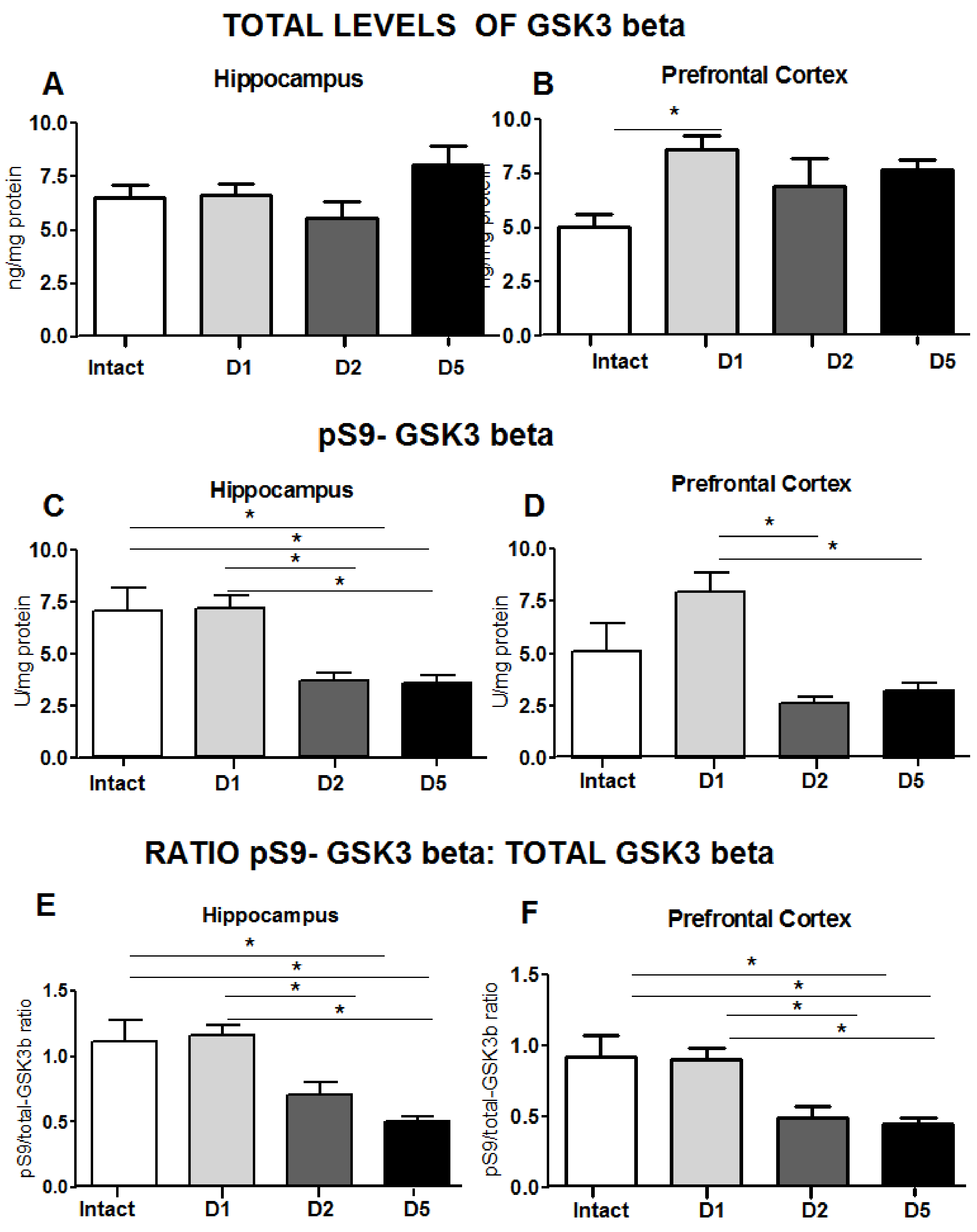

TOTAL LEVELS OF GSK3 beta
A
Hippocampus
ng/mg protein
10.0
7.5
5.0
2.5
0.0
Intact
D1
D2
D5
B
Prefrontal Cortex
*
pS9- GSK3 beta
C
Hippocampus
U/mg protein
D
Prefrontal Cortex
RATIO pS9- GSK3 beta: TOTAL GSK3 beta
E
Hippocampus
pS9/total-GSK3b ratio
1.5
1.0
0.5
0.0
F
Prefrontal Cortex

Figure 4

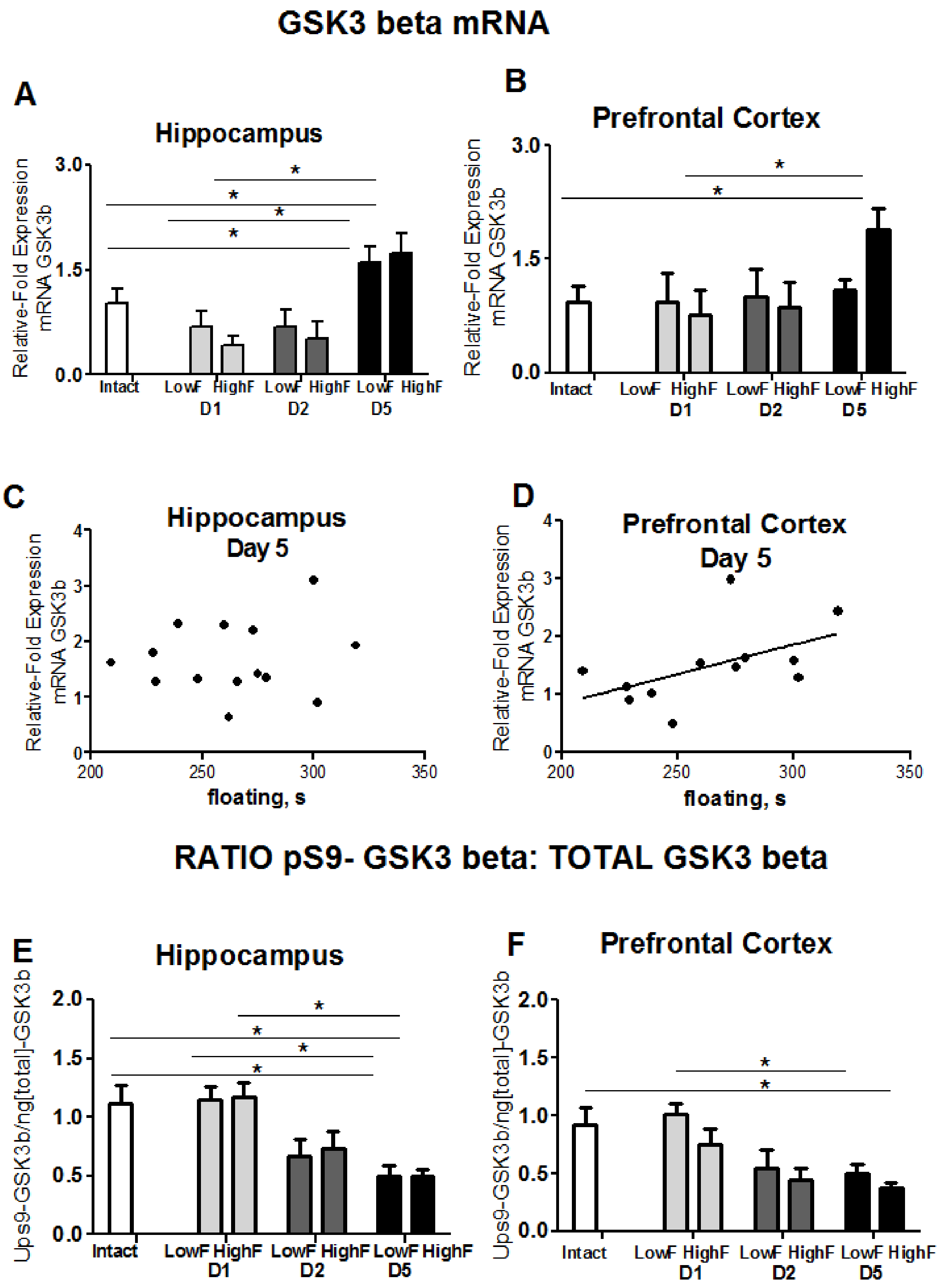
GSK3 beta mRNA
A
Hippocampus
Relative-Fold Expression mRNA GSK3b
3.0
1.5
0.0
Intact
LowF HighF LowF HighF LowF HighF
D1 D2 D5
B
Prefrontal Cortex
Relative-Fold Expression mRNA GSK3b
3.0
1.5
0.0
Intact
LowF HighF LowF HighF LowF HighF
D1 D2 D5
C
Hippocampus
Day 5
Relative-Fold Expression mRNA GSK3b
0 1 2 3 4
200 250 300 350
floating, s
D
Prefrontal Cortex
Day 5
Relative-Fold Expression mRNA GSK3b
0 1 2 3 4
200 250 300 350
floating, s
RATIO pS9- GSK3 beta: TOTAL GSK3 beta
E
Hippocampus
Ups9-GSK3b/ng[total]-GSK3b
2.0
1.5
1.0
0.5
0.0
Intact
LowF HighF LowF HighF LowF HighF
D1 D2 D5
F
Prefrontal Cortex
Ups9-GSK3b/ng[total]-GSK3b
2.0
1.5
1.0
0.5
0.0
Intact
LowF HighF LowF HighF LowF HighF
D1 D2 D5

Figure 5

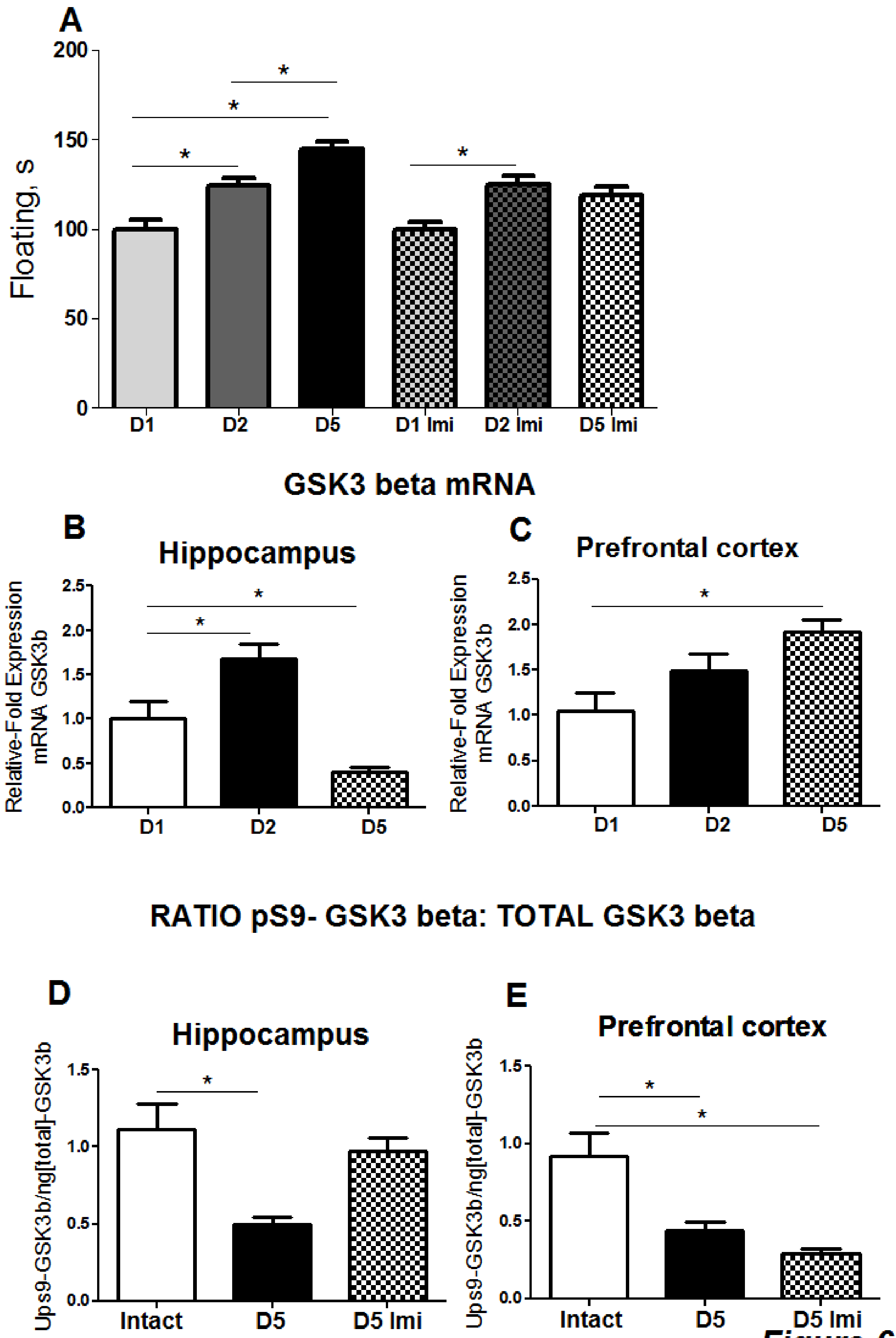
A
Floating, s
200
150
100
50
0
*
*
*
*
D1
D2
D5
D1 Imi
D2 Imi
D5 Imi
GSK3 beta mRNA
B
Hippocampus
Relative-Fold Expression mRNA GSK3b
2.5
2.0
1.5
1.0
0.5
0.0
*
*
D1
D2
D5
C
Prefrontal cortex
Relative-Fold Expression mRNA GSK3b
2.5
2.0
1.5
1.0
0.5
0.0
*
D1
D2
D5
RATIO pS9- GSK3 beta: TOTAL GSK3 beta
D
Hippocampus
Ups9-GSK3b/ng[total]-GSK3b
1.5
1.0
0.5
0.0
*
Intact
D5
D5 Imi
E
Prefrontal cortex
Ups9-GSK3b/ng[total]-GSK3b
1.5
1.0
0.5
0.0
*
*
Intact
D5
D5 Imi

Figure 6
